# In situ ruminal degradation kinetics of grass-legume mix baleage isoflavones in dairy cows fed diets with different forage:concentrate ratios

**DOI:** 10.3168/jdsc.2025-0939

**Published:** 2026-03-12

**Authors:** M.A. Arshad, M.G.M. Carvalho, R.A. Menezes, K.V. Almeida, O.R. Osuolale, M.M.H. Khandakar, W. Sims, M.A. Rahman, A.F. Brito

**Affiliations:** Department of Agriculture, Nutrition, and Food Systems, University of New Hampshire, Durham, NH 03824

## Abstract

Isoflavones are a class of phytoestrogens shown to have positive and negative effects on animal and human health. Red clover (RC) is a major source of isoflavones for dairy cows. We aimed to evaluate the in situ ruminal degradation kinetics of isoflavones from grass-legume (GR-LG) baleage composed of a mix of orchardgrass, white clover, and RC in lactating dairy cows fed diets with different forage:concentrate (F:C) ratios. Four ruminally cannulated multiparous Holstein cows averaging (mean ± SD) 214 ± 38.7 DIM, 741 ± 83.2 kg of BW, 23.1 ± 2.68 kg/d of DMI, and 35.7 ± 5.41 kg/d of milk yield in the beginning of the experiment were used in a crossover design study with 2 periods of 16 d each (14 d for diet adaptation and 2 d for sample incubation and collection). Cows were fed 2 experimental diets (DM basis): (1) low-forage (LF; 50:50 F:C ratio; 24.9% starch and 28.1% ash-free NDF), and (2) high-forage (HF; 70:30 F:C ratio; 20.8% starch and 33.4% ash-free NDF). Approximately 7 g of lyophilized and ground GR-LG baleage samples were weighed in triplicate nylon bags; sequentially incubated in the rumen of each cow in reverse order for 0 (not incubated in the rumen), 1, 2, 3, 4, 6, 8, 10, 12, 14, 16, 18, and 20 h; and simultaneously removed. The effective degradability (ED) of isoflavones in the rumen was calculated using the equation ED = *a* + *b* × [*K_d_*/(*K_d_* + *K_p_*)], where *a* is the soluble fraction (%), *b* is the potentially degradable fraction (%), *K_d_* is the rate of degradation of fraction *b* (%/h), and *K_p_* is the ruminal passage rate (%/h; 4.59 and 4.73 for HF and LF, respectively). The ruminal degradation of isoflavones was fit to the model $p=a+b\times \left(1-e^{\left(-K_{d}\times t\right)}\right),$ where *p* is the proportion (%) of isoflavones disappeared at time *t*, and *a*, *b*, and *K_d_* are as specified in the previous equation. The ED of total (93.3% vs. 92.5%) and individual isoflavones was greater in the HF diet than the LF diet. However, fractions *a* (52.7%), *b* (46.6%), and *K_d_* (30.4%/h) of total isoflavones were not affected by diets. Except for the ruminal disappearance (*p*) of genistein, which was greater in cows fed HF (85.5%) than those fed LF (81.1%), that of total isoflavones (87.1%), formononetin (87.4%), daidzein (80.6%), and biochanin A (86.5%) was not affected by treatments. In summary, our results showed that the ED of isoflavones from GR-LG baleage was greater when feeding HF compared with LF, but treatment differences were small and potentially not biologically meaningful.

## Highlights

• Isoflavones from grass-legume mix baleage degraded rapidly in the rumen.
• Cows fed the high-forage diet showed the greatest effective degradability of isoflavones.
• On average, over 80% of isoflavones disappeared after 20 hours of ruminal incubation.

## Graphical Abstract

Isoflavones are plant-derived compounds with estrogenic and antioxidant properties. This study examined the in situ ruminal degradation parameters of isoflavones from grass (orchardgrass)-legume (white and red clover) mix baleage in lactating dairy cows fed diets with varying forage:concentrate (F:C) ratios. Baleage samples were incubated in the rumen of cows receiving low-forage (50:50 F:C) or high-forage (70:30 F:C) diets. The isoflavones formononetin, genistein, biochanin A, and daidzein were extensively degraded in the rumen within 20 hours, irrespective of the diet. Cows fed the high-forage diet showed the greatest effective degradability of total and individual isoflavones.

**Figure:**
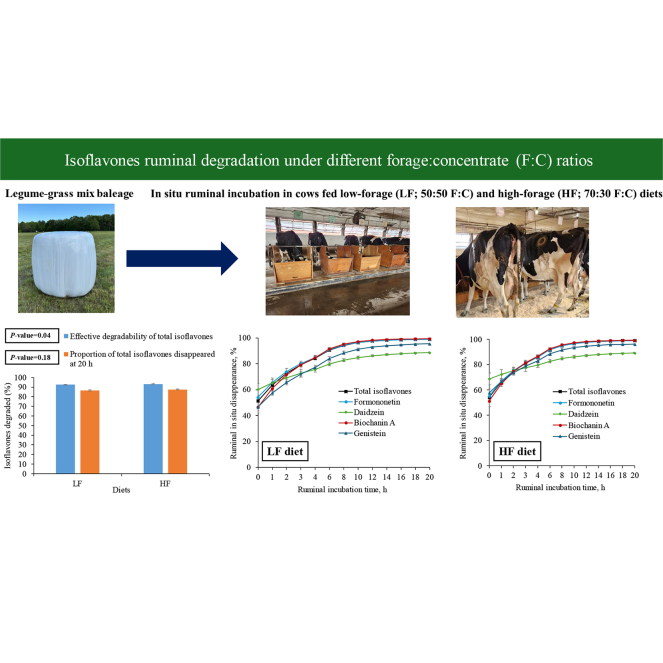

Isoflavones are a class of phytoestrogens with estrogenic and antioxidant properties (Wyse et al., 2022) found predominantly in red clover (**RC**; *Trifolium pratense* L*.*) and soybean (Flachowsky et al., 2011) and include compounds such as formononetin, biochanin A, daidzein, genistein, and prunetin (Mandal et al., 2024, 2025). Formononetin and biochanin A are the 2 major isoflavones present in RC (Mandal et al., 2024, 2025). Previous research showed that formononetin is primarily demethylated to daidzein by the ruminal microorganisms, followed by hydrogenation to generate equol (Nilsson et al., 1967; Dickinson et al., 1988). Equol is a phytoestrogen derivative with estrogenic activity as potent as genistein (COT, 2003) and is known to cause infertility in sheep and cattle (Wyse et al., 2022). Biochanin A is demethylated in the rumen to genistein and converted to nonestrogenic *p*-ethyl-phenol and organic acids (Nilsson et al., 1967; Batterham et al., 1971). Supplementation with synthetic biochanin A has been shown to increase milk yield and reduce the ruminal NH_3_-N concentration in dairy cows (Xiong et al., 2024; Bu et al., 2025), possibly because biochanin A impairs the growth of hyper-ammonia producing bacteria in the rumen (Flythe and Kagan, 2010). Isoflavones and equol are known to be transferred to milk (Njåstad et al., 2014), with their consumption associated with positive (e.g., decreased incidence of cardiovascular diseases and osteoporosis) and negative (e.g., hormone-dependent cancers) effects on human health (Sirotkin and Harrath, 2014). Therefore, understanding the ruminal degradation kinetics of isoflavones in lactating dairy cows is needed, given the potential role of these metabolites in modulating animal and human health (Sirotkin and Harrath, 2014) and modifying the gut microbiota profile in ruminants (Bu et al., 2025).

Isoflavones appears to be extensively degraded and metabolized in the rumen, with ruminal metabolism affected by their concentration in the diets, source of isoflavones, and dietary fiber concentration (Dickinson et al., 1988; Njåstad et al., 2014; Trnková et al., 2018). During an in situ study, Křížová et al. (2021) observed that isoflavones from solvent soybean meal and raw whole soybean were almost entirely degraded within 16 h of ruminal incubation in sheep fed a diet containing (DM basis) 60% grass hay and 40% concentrate. Data from Křížová et al. (2021) agree with those from Dickinson et al. (1988), Trnková et al. (2018), and Dadáková et al. (2020), who collectively showed a rapid and extensive in vitro degradation of soybean and RC isoflavones. However, we are not aware of any studies to date that have investigated the in situ ruminal degradation kinetics of isoflavones from grass-legume (**GR-LG**) baleage composed of a mix of orchardgrass (*Dactylis glomerata* L.), white clover (*Trifolium repens* L.), and RC in lactating dairy cows offered diets with varying forage:concentrate (**F:C**) ratios. Dadáková et al. (2020) reported lower concentrations of daidzein, formononetin, and biochanin A and a greater concentration of equol when comparing a hay-based diet and a concentrate-based diet in an in vitro study testing different levels of a RC extract, suggesting that diets rich in forages may favor isoflavones degradation and biotransformation in the rumen. In addition, diets with different F:C ratio have been shown to affect the rumen microbial community structure and fermentation characteristics in dairy cows (Wang et al., 2020; Ramos et al., 2021), which may ultimately impact the metabolism of isoflavones by the ruminal microbiota. Therefore, we aimed to measure the in situ ruminal degradation kinetics of GR-LG baleage isoflavones in lactating dairy cows fed diets with 2 levels of F:C ratios. Our hypothesis was that diets with different F:C ratios would change the ruminal fermentation profile ultimately affecting the degradation kinetics of GR-LG isoflavones.

This experiment was conducted at the University of New Hampshire Fairchild Dairy Teaching and Research Center (Durham, NH) from April to May 2024, with all procedures involving cows approved by the University Animal Care and Use Committee (protocol no. 240303). Four ruminally cannulated multiparous Holstein cows averaging (mean ± SD) 214 ± 38.7 DIM, 741 ± 83.2 kg of BW, 23.1 ± 2.68 kg/d of DMI, and 35.7 ± 5.41 kg/d of milk yield in the beginning of the experiment were used in a crossover design study with 2 experimental periods. Each period lasted 16 d, with 14 d for diet adaptation and 2 d for sample incubation and collection. Cows were fed 2 experimental diets (DM basis; Table 1): (1) low-forage (**LF**; 50:50 F:C ratio), and (2) high-forage (**HF**; 70:30 F:C ratio). After the last sampling on d 16 of period 1, ruminal digesta was exchanged between HF and LF cows to promote faster microbiota adaptation to the diet rotation (Agle et al., 2010). Cows were housed in a tiestall barn equipped with individual feed tubs and had free access to water throughout the experiment. Diets were formulated according to NASEM (2021) and fed ad libitum twice daily at 0430 h and 1530 h targeting 5% to 10% refusals. Samples of TMR, concentrate mixes, and refusals were collected during the last 5 d of each period, composited, dried in a forced-air oven (55°C, 48 h), and ground to pass through a 1-mm screen (Wiley mill, Arthur H. Thomas). Feeds were shipped to Dairy One Forage Testing Laboratory (Ithaca, NY) and analyzed for DM, CP, soluble CP, starch, ash-free NDF, ADF, ADL, and minerals via wet chemistry using the methods reported by Lange et al. (2024).

**Table 1:** Ingredient and chemical composition (% of DM, unless otherwise noted) of the experimental diets^1^

Item	LF	HF
Ingredient composition		
Corn silage	32.7	45.9
Legume-grass haylage	17.1	23.9
Energy mix^2^	30.6	11.0
Protein mix^3^	14.7	14.5
Amino enhancer^4^	1.43	1.41
Bergafat-100^5^	0.88	0.86
Mineral-vitamin premix	2.58	2.53
Chemical composition		
DM, % of fresh matter	41.1	38.5
CP	16.3	16.7
Soluble CP, % of CP	35.3	43.8
Starch	24.9	20.8
Ash-free NDF	28.1	33.4
ADF	19.7	23.6
ADL	3.08	3.43
Ca	1.24	0.83
P	0.39	0.41

1 Low forage (LF) = 50:50 forage:concentrate ratio; high forage (HF) = 70:30 forage:concentrate ratio.

2 Consisted of (as-fed basis): 45.8% ground corn, 35.2% beet pulp, 14.9% steam-flaked corn, and 4.10% sugarcane liquid molasses.

3 Consisted of (as-fed basis): 52.3% soybean meal, 23.5% SoyPlus (mechanically expelled soybean; RP Nutrients Inc.), 5.89% distillers dried grains with solubles, 17.5% canola meal, and 0.78% urea.

4 Consisted of (as-fed basis): 95.3% blood meal and 4.72% rumen-protected Met (Smartamine M, Adisseo USA Inc.).

5 BergaFat F100 (Berg+Schmidt America LLC) is a ruminally stable lipid supplement containing 80% palmitic acid.

The GR-LG baleage used in this experiment was produced at the University of New Hampshire Burley-Demeritt Organic Dairy Research Facility (Lee, NH). Briefly, first-cut GR-LG herbage was mowed on May 16, 2023, and harvested ~36 h later as round bales of baleage (n = 9), following the procedures reported by Resende et al. (2015). The botanical composition of the GR-LG sward (0.54 ha) was determined according to Brito et al. (2017) and Isenberg et al. (2019), with herbage samples separated (DM basis) into grass (53.7% mostly orchardgrass), legumes (39.4% = 21.9% white clover and 17.5% RC), and weeds (6.92%). An electric drill (model TE 7-A, Hilti Inc.) fitted with a 45-cm stainless steel core sampler barrel (Nasco) was used to sample (∼200 g) the 9 bales, with collected materials placed in a cooler with dry ice and stored at −20°C. Next, baleage samples were lyophilized for 48 h, ground to pass through a 1-mm screen, and shipped to the North Dakota State University Veterinary Diagnostic Laboratory (Fargo, ND) for analysis of isoflavones. Samples were extracted in methanol:water solution (80:20), followed by sonication and enzymatic hydrolysis of glucosylated isoflavones to their aglycone forms using β-glucosidase, and then quantified by liquid chromatography coupled with triple-quadrupole mass spectrometry. The 9 GR-LG bales were ranked based on the total isoflavones concentration, which ranged (DM basis) from 370 to 1,689 mg/kg, and the bale with the greatest phytoestrogens level (i.e., 1,689 mg/kg) was selected to be used in the study. The selected bale was chopped using a vertical mixer (Valmetal V-Mix 400), with forage materials from different locations within the pile collected and pooled to obtain a representative baleage sample (~6.0 kg) that was stored at −20°C until lyophilization.

A subsample of lyophilized baleage was ground (1 mm) and shipped to the North Dakota State University Veterinary Diagnostic Laboratory for analysis of isoflavones, as previously described. A second subsample of lyophilized baleage was ground (5 mm), weighed (~7 g) in triplicate into nylon bags (10 × 20 cm) with 50 ± 10 μm porosity (ANKOM Technology Corp.), and closed with zip ties. These nylon bags were placed in larger laundry mesh bags (36 × 42 cm); sequentially incubated in the rumen of each cow on d 16 of each sampling period in reverse order (i.e., from the longest to the shortest time point) for 1, 2, 3, 4, 6, 8, 10, 12, 14, 16, 18, and 20 h; and simultaneously removed by time point. The 0-h time point bags (n = 3/cow) were not ruminally incubated, but were processed in the same manner as the incubated bags following removal from the rumen to determine the instantaneously soluble fraction. All bags were lyophilized, opened, and residues were analyzed for isoflavones, as done for the baleage samples. The in situ effective degradability (**ED**) of isoflavones in the rumen was calculated using the following equation (Ørskov and McDonald, 1979): ED = *a* + *b* × [*K_d_*/(*K_d_* + *K_p_*)], where *a* is the soluble fraction (%), *b* is the potentially degradable fraction (%), *K_d_* is the degradation rate of fraction *b* (%/h), and *K_p_* is the ruminal passage rate, which was estimated at 4.59%/h and 4.73%/h for the HF and LF diet, respectively, according to Seo et al. (2006). Specifically, *K_p_* was calculated using the following equation: *K_p_* = [2.365 + 0.0214 × forage DMI (g/kg of BW) + 0.0734 × concentrate DMI (g/kg of BW) + 0.069 × forage DMI (kg/d)]/100. Forage and concentrate DMI values were estimated based on actual DMI and ingredient composition of the experimental diets. Degradation curves of isoflavones were fitted to the Ørskov and McDonald (1979) model as follows: $p=a+b\times \left(1-e^{\left(-K_{d}\times t\right)}\right),$ where *p* is the proportion (%) of isoflavones degraded or disappeared at time *t*, and *a*, *b*, and *K_d_* are defined as described previously.

Samples of ruminal digesta (~200 mL) were taken from the ventral sac of the rumen of the 4 ruminally cannulated cows using a hand pump fitted with a metal dipper on d 15 of each sampling period at 0 h (immediately before the 0530 h feeding), and at 1, 2, 4, 6, 8, 10, 12, 14, 16, and 18 h after feeding. Next, samples were strained through 2 layers of cheesecloth, with filtered ruminal fluid transported to the laboratory and recorded for pH using a portable pH meter (model SP20, VWR International). Following pH measurements, 20-mL subsamples were collected, preserved with 0.4 mL of 50% H_2_SO_4_ (vol/vol) in 50-mL vials, stored at −20°C, and shipped to Dairy One Forage Testing Laboratory for determination of VFA through GC (Perkin Elmer Inc.) and NH_3_-N via diffusion (Timberline TL-2800 analyzer).

In situ ruminal degradation (*a*, *b*, *K_d_*, and *p*) data were fitted to the NLIN procedure of SAS (version 9.4; SAS Institute Inc.) using the Marquardt algorithm to obtain the smallest residual sum of squares and analyzed with the MIXED (total VFA concentration, *a*, *b*, *K_d_*, and ED) or GLIMMIX (ruminal individual VFA and NH_3_-N, and *p*) procedure of SAS according to a crossover design. Sequence (order in which treatments were administered to the same experimental unit [cow]), period, and treatment were entered as fixed effects in the model, with cow nested within sequence and the residual error as random terms. All ruminal fermentation data and *p* were analyzed using repeated measures over time, with time of sampling as the repeated term and the heterogeneous autoregressive (1) covariance matrix retained in the final models based on the lowest Bayesian information criterion. Statistical significance was declared at *P* ≤ 0.05 and tendencies at 0.05 < *P* ≤ 0.10, with the PDIFF procedure of SAS used to separate treatment means.

The GR-LG baleage used in our study averaged 50.1% DM (% of fresh matter) and contained (DM basis): 18.1% CP, 12.3% soluble CP, 43.5% ash-free NDF, 32.9% ADF, and 5.50% ADL. It also contained (DM basis) 1,518 mg/kg total isoflavones (calculated as the sum of individual isoflavones), fractionated into 941 mg/kg formononetin, 470 mg/kg biochanin A, 79.0 mg/kg genistein, and 28.0 mg/kg daidzein. Glycitein and coumestrol concentrations were below the instrument detection limits of <10 and <5 mg/kg of DM, respectively. We measured ruminal pH and concentrations of VFA and NH_3_-N to characterize the fermentation profile in response to diets while acknowledging the limited number of observations per treatment to detect statistical differences. However, diet × time interactions (*P* ≤ 0.01) were observed for the ruminal concentrations of total VFA and NH_3_-N (data not shown). Specifically, the total VFA concentration was lower (*P* < 0.001) in HF than LF (107 vs. 128 m*M*; SEM = 8.81 m*M*) at 10 h after the morning feeding, and that of NH_3_-N was greater (*P* ≤ 0.02) in HF compared with LF at 8 h (5.72 vs. 1.61 mg/dL; SEM = 1.43 mg/dL), 14 h (14.5 vs. 4.94 mg/dL; SEM = 2.01 mg/dL), and 16 h (10.5 vs. 2.90 mg/dL; SEM = 1.98 mg/dL) after feeding. We also observed that the ruminal molar proportion of butyrate tended (*P* = 0.06) to be lower in cows fed HF than LF (9.70% vs. 11.0%; SEM = 0.34%). Contrarily, ruminal pH (5.94 vs. 6.00; SEM = 0.13) and the molar proportions of acetate (65.7% vs. 67.4%; SEM = 0.19%) and propionate (21.6% vs. 19.8%; SEM = 1.11%) were not affected (*P* ≥ 0.19) with feeding LF versus HF.

The in situ ED of GR-LG baleage total isoflavones (93.3% vs. 92.5%), formononetin (93.2% vs. 92.7%), daidzein (85.5 vs. 83.4%), and biochanin A (93.3% vs. 92.5%) were all greater (*P* ≤ 0.04) in cows fed HF than LF, and that of genistein (90.5% vs. 87.9%) tended (*P* = 0.07) to be greater with feeding the HF versus the LF diet (Table 2). Despite increased isoflavones ED with feeding the HF diet, differences between treatments were small (ranging from 0.8 to 2.6 percentage units) and likely of limited biological relevance. Nevertheless, the *K_d_* of formononetin tended (*P* = 0.08) to be greater in HF than LF cows (31.2%/h vs. 28.7%/h), partially explaining the increase in formononetin ED. Furthermore, increased ED of daidzein can be attributed to a slightly greater treatment difference for fraction *a* (68.7% vs. 60.1%; *P* = 0.04) than fraction *b* (28.7% vs. 21.2%; *P* = 0.06) in cows receiving the HF versus the LF diet, as *K_d_* did not differ between diets. Note that the greater ED of total isoflavones, biochanin A, and genistein in HF versus LF was not accompanied by changes in fractions *a* and *b*, or *K_d_*. The mean ED of daidzein (84.5%) and genistein (89.2%) was lower than that of formononetin (93.0%) and biochanin A (92.9%), suggesting differences in the ruminal metabolism of these isoflavones.

**Table 2:** Ruminal degradability of isoflavones from grass-legume mix baleage in dairy cows fed diets with different forage:concentrate (F:C) ratios

Item^2^	Diet^1^		SEM	*P*-value		
	LF	HF		Diet	Time	Diet × time
Total isoflavones						
ED, %	92.5	93.3	0.44	0.04	—	—
*a*, %	51.3	54.0	2.28	0.33	—	—
*b*, %	47.9	45.2	2.33	0.35	—	—
*K_d_*, %/h	29.3	31.3	3.74	0.13	—	—
*p*, %	86.5	87.6	0.48	0.18	<0.01	0.82
Formononetin						
ED, %	92.7	93.2	0.31	0.04	—	—
*a*, %	54.0	54.9	2.84	0.77	—	—
*b*, %	45.0	44.1	2.91	0.76	—	—
*K_d_*, %/h	28.7	31.2	3.15	0.08	—	—
*p*, %	87.1	87.7	1.02	0.68	0.05	0.99
Daidzein						
ED, %	83.4	85.5	0.60	0.02	—	—
*a*, %	60.1	68.7	1.16	0.04	—	—
*b*, %	28.7	21.2	1.48	0.06	—	—
*K_d_*, %/h	18.7	18.1	2.10	0.81	—	—
*p*, %	79.1	82.2	1.73	0.21	0.11	0.61
Biochanin A						
ED, %	92.5	93.3	0.36	0.03	—	—
*a*, %	47.7	51.0	0.66	0.14	—	—
*b*, %	51.6	48.2	0.72	0.15	—	—
*K_d_*, %/h	32.3	33.8	2.62	0.13	—	—
*p*, %	86.2	87.5	2.01	0.46	0.01	0.99
Genistein						
ED, %	87.9	90.5	0.89	0.07	—	—
*a*, %	49.2	56.8	1.15	0.12	—	—
*b*, %	46.6	39.6	1.22	0.05	—	—
*K_d_*, %/h	24.4	26.5	3.35	0.31	—	—
*p*, %	81.1	85.5	1.18	0.01	0.01	0.08

1 Low forage (LF) = 50:50 F:C ratio; high-forage (HF) = 70:30 F:C ratio.

2 ED = effective degradability; *a* = soluble fraction; *b* = potentially degradable fraction; *K_d_* = degradation rate of fraction *b*; *p* = proportion of isoflavones degraded.

Křížová et al. (2021) reported greater in situ *K_d_* (53.0%/h) and fraction *b* (49.8%) and lower fraction *a* (50.1%) for solvent-extracted soybean meal total isoflavones in sheep fed (DM basis) 60% grass hay and 40% concentrate compared with LG-GR baleage total isoflavones *K_d_* (30.4%/h), fraction *b* (46.6%), and fraction *a* (52.7%) averaged across diets (Table 2). However, Křížová et al. (2021) showed that the *K_d_* (35.4%/h) and fraction *a* (53.4%) of ground raw whole soybean total isoflavones were similar to the *K_d_* and fraction *a* of GR-LG total isoflavones. Therefore, the differences in ruminal degradation parameters between solvent-extracted soybean meal and GR-LG may be associated with the type of feed incubated in the rumen (concentrate vs. forage), interference of the solvent used to extract oil from whole soybean before generating soybean meal, and the animal species (sheep vs. cows) enrolled in the in situ studies. Furthermore, the initial concentration of total isoflavones could have also played a role because it was 60.1% and 67.2% greater in solvent-extracted soybean meal (2,441 mg/kg of DM) than GR-LG baleage (1,518 mg/kg of DM) and ground raw whole soybean (1,460 mg/kg of DM), respectively.

The proportion of total isoflavones, formononetin, daidzein, and biochanin A degraded in the rumen was not affected (*P* ≥ 0.18) by diets (Table 2). In addition, no diet × time interactions (*P* ≥ 0.08) were observed for the ruminal disappearance of isoflavones. Therefore, we are presenting the isoflavones degradation curves separated by diet (Figure 1). After 20 h of ruminal incubation, the ruminal disappearance of total isoflavones reached up to 99.0%, regardless of the diet fed to the cows (Figure 1). Shutt et al. (1970) showed that the abomasal flow of total isoflavones averaged 670 mg/d in Merino wethers fed 1 kg/d of dried RC herbage, corresponding to a daily intake of 9,200 mg of total isoflavones. These results revealed that 92.7% of the total isoflavones consumed were metabolized or absorbed in the sheep's forestomach. Additionally, 81.6% of formononetin (5,500 mg/d) and daidzein (100 mg/d) ingested by the wethers were converted into equol in the gastrointestinal tract, with 650 mg/d of equol flowing to the small intestine and 3,700 and 220 mg/d excreted in the urine and feces, respectively (Shutt et al., 1970). Shutt et al. (1970) also reported that the concentrations of formononetin, daidzein, and biochanin A in the liquid phase of the ruminal digesta decreased by 90.8%, 59.4%, and 93.6% between 30 and 90 min after feeding, respectively. This suggests rapid degradation of isoflavones in the rumen, metabolism to other compounds, absorption through the ruminal wall, or flow to the small intestine. Njåstad et al. (2014) observed that only a small proportion of ingested isoflavones (ranging from 0.49% [genistein] to 10.3% [daidzein]; average = 2.76%) escaped ruminal degradation and appeared in the small intestine of lactating dairy cows fed timothy grass (*Phleum pratense* L.) and RC-based silage, showing extensive metabolism or absorption of these isoflavones in the rumen. Equol accounted for 84.7% of the total amount of isoflavones leaving the omasum, with the digestive tract serving as the major excretory route for equol in dairy cows (Njåstad et al., 2014). Overall, Shutt et al. (1970) and Njåstad et al. (2014) demonstrated that isoflavones from RC were extensively metabolized and absorbed in the rumen, with their findings further supported by our in situ results.

**Figure 1:**
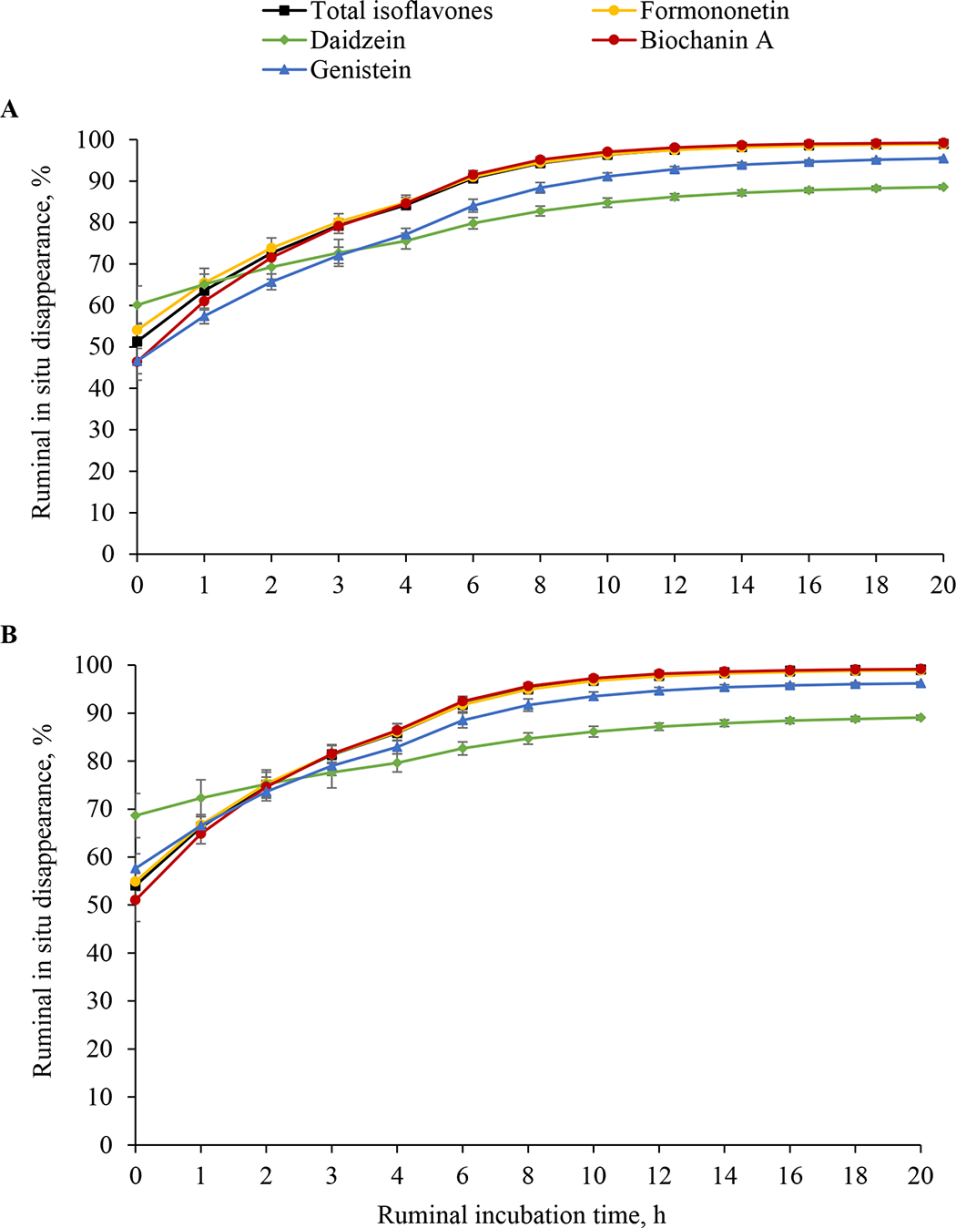
Ruminal in situ disappearance of total and individual isoflavones from grass-legume mix baleage incubated in dairy cows fed diets with different forage:concentrate (F:C) ratios: (A) low-forage diet (50:50 F:C ratio); (B) high-forage diet (70:30 F:C ratio). Error bars indicate SEM.

In contrast to the other individual isoflavones discussed previously, the proportion of genistein degraded in the rumen was greater (*P* = 0.01) in cows fed HF (85.5%) than LF (81.1%) diets (Table 2). Dadáková et al. (2020) reported greater in vitro degradation of formononetin, daidzein, and biochanin A from various amounts (25–75 mg) of RC extract incubated (up to 24 h) in ruminal fluid obtained from nonlactating, ruminally cannulated cows fed hay versus a diet with 65:35 F:C ratio. However, the in vitro degradation of genistein from RC extract was not affected by the source of ruminal fluid included in the incubations. We observed that among all individual isoflavones evaluated, daidzein exhibited the lowest ruminal disappearance after 20 h of in situ incubation averaging 80.7% across diets. This may be explained by daidzein's longer half-life (9.8 h) in ruminal fluid compared with that of formononetin (4.3 h), biochanin A (3.9 h), and genistein (5.5 h) based on the in vitro study of Dickinson et al. (1988). Similarly, Shutt et al. (1970) and Njåstad et al. (2014), through in vivo studies conducted with sheep and cows, respectively, along with Dadáková et al. (2020), in an in vitro experiment, demonstrated that despite daidzein being extensively metabolized by the ruminal microbiota, it was more resistant to degradation relative to other isoflavones present in RC and soybean. Although the factor(s) associated with the stronger resistance of daidzein to ruminal metabolism are not well understood, it is conceivable that daidzein may be less absorbed and biotransformed in the rumen than other RC isoflavones.

In summary, our results showed that the ED of GR-LG baleage total and individual isoflavones was greater with feeding the HF than the LF diet to lactating dairy cows. However, treatment differences for isoflavones ED were small and possibly not biologically relevant. We also showed that the ruminal degradation or disappearance of GR-LG baleage isoflavones was extensive, averaging over 80% across metabolites, diets, and time points.

## References

[bib1] Agle M., Hristov A.N., Zaman S., Schneider C., Ndegwa P., Vaddella V. (2010). Effect of dietary concentrate on rumen fermentation, digestibility, and nitrogen losses in dairy cows. J. Dairy Sci..

[bib2] Batterham T.J., Shutt D.A., Hart N.K., Braden A.W.H., Tweeddale H.J. (1971). Metabolism of intraruminally administered [4-^14^C] formononetin and [4-^14^C] biochanin A in sheep. Aust. J. Agric. Res..

[bib3] Brito A.F., Soder K.J., Chouinard P.Y., Reis S.F., Ross S., Rubano M.D., Casler M.D. (2017). Production performance and milk fatty acid profile in grazing dairy cows offered ground corn or liquid molasses as the sole supplemental nonstructural carbohydrate source. J. Dairy Sci..

[bib4] Bu Y., Zhang X., Xiong Z., Li K., Zhang S., Lin M., Zhao G., Zheng N., Wang J., Zhao S. (2025). Effect of red clover isoflavones on ruminal microbial composition and fermentation in dairy cows. Appl. Microbiol. Biotechnol..

[bib5] COT (Committee on Toxicity of Chemicals in Food) (2003). https://webarchive.nationalarchives.gov.uk/ukgwa/20200803163735/https://cot.food.gov.uk/cotreports/cotwgreports/phytoestrogensandhealthcot.

[bib6] Dadáková K., Trnková A., Kašparovská J., Křížová L., Lochman J., Kašparovský T. (2020). In vitro metabolism of red clover isoflavones in rumen fluid. J. Anim. Physiol. Anim. Nutr. (Berl.).

[bib7] Dickinson J.M., Smith G.R., Randel R.D., Pemberton I.J. (1988). In vitro metabolism of formononetin and biochanin A in bovine rumen fluid. J. Anim. Sci..

[bib8] Flachowsky G., Hünerberg M., Meyer U., Kammerer D.R., Carle R., Goerke M., Eklund M. (2011). Isoflavone concentration of soybean meal from various origins and transfer of isoflavones into milk of dairy cows. J. Verbrauch. Lebensm..

[bib9] Flythe M., Kagan I. (2010). Antimicrobial effect of red clover (*Trifolium pratense*) phenolic extract on the ruminal hyper ammonia-producing bacterium, *Clostridium sticklandii*. Curr. Microbiol..

[bib10] Isenberg B.J., Soder K.J., Pereira A.B.D., Standish R., Brito A.F. (2019). Production, milk fatty acid profile, and nutrient utilization in grazing dairy cows supplemented with ground flaxseed. J. Dairy Sci..

[bib11] Křížová L., Němcová Z., Dadáková K., Chrenková M. (2021). In sacco evaluation of ruminal degradability of isoflavones from full-fat soybean and extracted soybean meal—a pilot study. J. Anim. Physiol. Anim. Nutr. (Berl.).

[bib12] Lange M.J., Silva L.H.P., Zambom M.A., Soder K.J., Brito A.F. (2024). Feeding alfalfa-grass or red clover–grass mixture baleage: Effect on milk yield and composition, ruminal fermentation and microbiota taxa relative abundance, and nutrient utilization in dairy cows. J. Dairy Sci..

[bib13] Mandal P., Lima M.R., Wallingford A.K., Warren N.D., Brito A.F., Smith R.G. (2025). Short-term exposure to elevated temperature and CO_2_ alters phytoestrogen production in red clover. Sci. Rep..

[bib14] Mandal P., Mortensen D.A., Brito A.F., Wallingford A.K., Lima M.R., Warren N.D., Smith R.G. (2024). Water stress influences phytoestrogen levels in red clover (*Trifolium pratense*) but not Kura clover (*T. ambiguum*). J. Agric. Food Chem..

[bib15] NASEM. (National Academies of Sciences, Engineering, and Medicine) (2021). https://doi.org/10.17226/25806.

[bib16] Nilsson A., Hill J., Lloyd Davies H. (1967). An in vitro study of formononetin and biochanin A metabolism in rumen fluid from sheep. Biochim. Biophys. Acta.

[bib17] Njåstad K.M., Adler S.A., Hansen-Møller J., Thuen E., Gustavsson A.-M., Steinshamn H. (2014). Gastrointestinal metabolism of phytoestrogens in lactating dairy cows fed silages with different botanical composition. J. Dairy Sci..

[bib18] Ørskov E.R., McDonald I. (1979). The estimation of protein degradability in the rumen from incubation measurements weighted according to rate of passage. J. Agric. Sci..

[bib19] Ramos S.C., Jeong C.D., Mamuad L.L., Kim S.H., Kang S.H., Kim E.T., Cho Y.I., Lee S.S., Lee S.S. (2021). Diet transition from high-forage to high-concentrate alters rumen bacterial community composition, epithelial transcriptomes, and ruminal fermentation parameters in dairy cows. Animals (Basel).

[bib20] Resende T.L., Kraft J., Soder K.J., Pereira A.B.D., Woitschach D.E., Reis R.B., Brito A.F. (2015). Incremental amounts of ground flaxseed decrease milk yield but increase n-3 fatty acids and conjugated linoleic acids in dairy cows fed high-forage diets. J. Dairy Sci..

[bib21] Seo S., Tedeschi L.O., Lanzas C., Schwab C.G., Fox D.G. (2006). Development and evaluation of empirical equations to predict feed passage rate in cattle. Anim. Feed Sci. Technol..

[bib22] Shutt D., Weston R., Hogan J. (1970). Quantitative aspects of phyto-oestrogen metabolism in sheep fed on subterranean clover (*Trifolium subterraneum* cultivar Clare) or red clover (*Trifolium pratense*). Aust. J. Agric. Res..

[bib23] Sirotkin A.V., Harrath A.H. (2014). Phytoestrogens and their effects. Eur. J. Pharmacol..

[bib24] Trnková A., Šancová K., Zapletalová M., Kašparovská J., Dadáková K., Křížová L., Lochman J., Hadrová S., Ihnatová I., Kašparovský T. (2018). Determination of in vitro isoflavone degradation in rumen fluid. J. Dairy Sci..

[bib25] Wang L., Li Y., Zhang Y., Wang L. (2020). Effects of different concentrate-to-forage ratio diets on rumen bacterial microbiota and community structure in Holstein cows during the feeding cycle. Animals (Basel).

[bib26] Wyse J., Latif S., Gurusinghe S., McCormick J., Weston L.A., Stephen C.P. (2022). Phytoestrogens: A review of their impacts on reproductive physiology and other effects upon grazing livestock. Animals (Basel).

[bib27] Xiong Z., Li Y., Zhang X., Zhang S., Li K., Zheng N., Zhao S., Wang J. (2024). Effects of biochanin A on lactational performance, nitrogen metabolism, and blood metabolites in dairy cows. Anim. Nutr..

